# Cost-effectiveness analysis of sorafenib, lenvatinib, atezolizumab plus bevacizumab and sintilimab plus bevacizumab for the treatment of advanced hepatocellular carcinoma in China

**DOI:** 10.1186/s12962-023-00435-x

**Published:** 2023-03-31

**Authors:** Hongyu Gong, Siew Chin Ong, Fan Li, Zhiying Weng, Keying Zhao, Zhengyou Jiang

**Affiliations:** 1 School of Pharmaceutical Sciences, Universiti Sains Malaysia, 11800 USM, Penang Malaysia; 2 Incubation Center for Scientific and Technological Achievements, Kunming Medical University, Chunrong West Road 1168, Kunming City, China; 3 School of Pharmaceutical Science &Yunnan Key Laboratory of Pharmacology for Natural Products, Kunming Medical University, Chunrong West Road 1168, Kunming City, China; 4 School of Public Health, Kunming Medical University, Chunrong West Road 1168, Kunming City, China; 5 School of Management, Universiti Sains Malaysia, 11800 USM Penang City, Penang Malaysia; 6 Yunnan Drug Policy Research Center, Kunming Medical University, Kunming, China

**Keywords:** Cost-effectiveness analysis, Sorafenib, Lenvatinib, Atezolizumab plus bevacizumab, Sintilimab plus bevacizumab, Advanced hepatocellular carcinoma, China

## Abstract

**Background and Objective:**

Hepatocellular carcinoma (HCC) is one of the leading causes of cancer-related death worldwide, especially in China. According to the 2021 Chinese Society of Clinical Oncology guidelines, sorafenib, lenvatinib, atezolizumab combined with bevacizumab, and sintilimab combined with bevacizumab are recommended as first-line treatment options for advanced HCC. This study provides a cost-effectiveness analysis of these treatments from the patient perspective.

**Methods:**

A partitioned survival model was established using the TreeAge 2019 software to evaluate the cost-effectiveness. The model includes three states, namely progression-free survival, progressive disease, and death. Clinical data were derived from three randomized controlled studies involving patients with advanced HCC who received the following treatment: sorafenib and lenvatinib (NCT01761266); atezolizumab in combination with bevacizumab (NCT03434379); and sintilimab in combination with bevacizumab (NCT03794440). Cost and clinical preference data were obtained from the literature and interviews with clinicians.

**Results:**

All compared with sorafenib therapy, lenvatinib had an incremental cost-effectiveness ratio (ICER) of US$188,625.25 per quality-adjusted life year (QALY) gained; sintilimab plus bevacizumab had an ICER of US$75,150.32 per QALY gained; and atezolizumab plus bevacizumab had an ICER of US$144,513.71 per QALY gained. The probabilistic sensitivity analysis indicated that treatment with sorafenib achieved a 100% probability of cost-effectiveness at a threshold of US$36,600/QALY. One-way sensitivity analysis revealed that the results were most sensitive to the medical insurance reimbursement ratio and drug prices.

**Conclusions:**

In this economic evaluation, therapy with lenvatinib, sintilimab plus bevacizumab, and atezolizumab plus bevacizumab generated incremental QALYs compared with sorafenib; however, these regimens were not cost-effective at a willingness-to-pay threshold of US$36,600 per QALY. Therefore, some patients may achieve preferred economic outcomes from these three therapies by tailoring the regimen based on individual patient factors.

**Supplementary Information:**

The online version contains supplementary material available at 10.1186/s12962-023-00435-x.

## Key points

With the approval of new first-and second-line drugs and the establishment of treatment based on immune checkpoint inhibitors as standard treatment, treatment options for advanced hepatocellular carcinoma are more diverse than ever before. Therefore, clinical decision-making requires a multidisciplinary team to develop individualized treatment strategies according to the patients' disease and financial ability to pay. Here, we point out that lenvatinib, sintilimab plus bevacizumab and atezolizumab plus bevacizumab are more effective in the treatment of advanced hepatocellular carcinoma, but the cost of treatment is beyond the affordability of the patient. We outlined better drug purchase prices and health insurance reimbursement policies to enable patients to get the optimal treatment.

**Supplementary Information:**

The online version contains supplementary material available at 10.1186/s12962-023-00435-x.

## Introduction

China ranks fifth and second worldwide in terms of the incidence and mortality, respectively, of liver cancer or hepatocellular carcinoma (HCC) [1] Due to the low awareness among individuals concerning preventive medicine, a considerable number of patients are diagnosed with HCC at an advanced stage. Consequently, these patients have missed the opportunity for some local treatments, such as hepatectomy, local ablation, hepatic artery intervention, and radiation therapy [2, 3].

Chemotherapy is an option for patients with advanced or metastatic HCC; however, the effect of this treatment approach on the prolongation of survival is limited [4, 5]. Sorafenib is the first multi-targeted drug that has shown effectiveness in prolonging the survival of patients with advanced HCC [6]. It inhibits vascular endothelial growth factors (VEGFs), Platelet-derived growth factor (PDGF), and serine/threonine kinase. The SHARP study showed that the overall survival (OS) of patients in the sorafenib group was markedly longer than that recorded in the placebo group (10.7 and 7.9 months, respectively, hazard ratio [HR]: 0.69; 95% confidence interval [CI] 0.55–0.87) [6]. Since 2018, the treatment options for patients with advanced HCC have increased. For example, it has been demonstrated that treatment with lenvatinib, programmed cell death 1 ligand 1 (PD-L1) inhibitor atezolizumab combined with bevacizumab, and programmed cell death 1 (PD-1) inhibitor sintilimab combined with bevacizumab, is effective against advanced HCC. The NCT01761266 trial showed that lenvatinib was non-inferior to sorafenib in terms of OS (13.6 and 12.3 months, respectively, HR: 0.92; 95% CI: 0.79–1.06) [7]. In the NCT03434379 study, the rate of 12-month OS in the atezolizumab combined with bevacizumab group and the sorafenib group was 67.2% and 54.6%, respectively (HR: 0.58; 95% CI: 0.42–0.79) [8]. In the NCT03794440 trial, sintilimab was linked to a markedly longer OS than sorafenib (median not reached and 10.4 months, respectively, HR: 0.57; 95% CI: 0.43–0.75) [9]. Therefore, direct evidence shows that atezolizumab combined with bevacizumab and sintilimab combined with bevacizumab are better than sorafenib alone in terms of therapeutic effect. However, there is limited evidence on the cost-effectiveness of these four treatment options from the patient perspective. The objective of the present analysis was to investigate the cost-effectiveness of these four therapies as a first-line therapy for advanced HCC from the patient perspective in China.

## Methods

### Analytical overview

A partitioned survival model was constructed to simulate the disease process of advanced HCC and to estimate the cost-effectiveness of sorafenib, lenvatinib, atezolizumab plus bevacizumab, and sintilimab plus bevacizumab for patients in China.

Patients with advanced HCC or unresectable tumors without prior systemic therapy were selected for inclusion in the model. When patients with advanced hepatocellular carcinoma progress after receiving the first-line treatment, the physician will choose to stop the first-line treatment and start using the second-line drugs. In the model, regorafenib was selected as the second-line treatment plan for progressive hepatocellular carcinoma because regorafenib was the most commonly used treatment in clinical practice. Based on disease progression, three transition states of disease were defined: progression-free survival (PFS); progressive disease (PD); and death. The cohort flow was determined by survival curves over time. Each survival curve described the movement of patients out of the health state associated with that curve and into the next state of progression. At a certain time (t), the survival ratio of PD was equal to the OS value minus the PFS value. The proportions of patients with PFS and OS were calculated based on the results of the NCT01761266, NCT03434379, and NCT03794440 trials. The evidence was validated by comparing the PFS and OS results of the model with the observed and extrapolated data [6–8, 10, 11]. The length of each partitioned survival model cycle was 1 month, and survival was adjusted for quality of life based on specific utilities. Direct fees paid by patients at the hospital included medicine drugs, bed fees, testing fees, and adverse reaction processing fees. According to the 2020 edition of the “Guidelines for the Evaluation of Chinese Pharmacoeconomics”, all fees and utilities are discounted by 5% [12]. Models were constructed using the TreeAge Pro 2019 software (2019 TreeAge Software, Inc.).

### Efficacy and safety input

NCT01761266 was a multicenter, randomized, open-label trial. It compared the efficacy and safety of lenvatinib versus sorafenib as first-line systemic therapy for unresectable HCC. A total of 954 patients were randomized in a 1:1 ratio to receive lenvatinib or sorafenib. The study included patients aged ≥ 18 years, diagnosed with advanced HCC, and categorized as Barcelona Clinic Liver Cancer stage B or C and Child–Pugh class A. More information on the inclusion and exclusion criteria is available elsewhere [7].

NCT03434379 was a global, randomized, open-label study. It compared the efficacy and safety of atezolizumab-bevacizumab versus sorafenib as first-line systemic therapy for unresectable HCC. Patients aged ≥ 18 years with locally advanced metastatic or unresectable HCC (or both) and those with advanced HCC who had not received prior systemic therapy were enrolled. More information on the inclusion and exclusion criteria is available elsewhere [8]. The patients were randomized in a 2:1 ratio to receive atezolizumab plus bevacizumab or sorafenib until the occurrence of intolerable toxicity or disease progression. The intent-to-treat population included 336 and 165 patients in the atezolizumab-bevacizumab and sorafenib groups, respectively.

NCT03794440 was randomized, open-label, multicenter study conducted in China. It compared the efficacy and safety of sintilimab-bevacizumab versus sorafenib as first-line systemic therapy for unresectable HCC. Patients aged ≥ 18 years with histologically or cytologically diagnosed or clinically confirmed unresectable or metastatic HCC, no prior systemic therapy, and baseline Eastern Cooperative Oncology Group performance status of 0 or 1 were eligible for selection. A total of 595 patients were randomly assigned in a 2:1 ratio to receive sintilimab plus bevacizumab or sorafenib until disease progression or the occurrence of unacceptable toxicity. More information on the inclusion and exclusion criteria is available elsewhere [9].

In these studies, lenvatinib, atezolizumab plus bevacizumab, and sintilimab plus bevacizumab shared a common comparator (i.e., sorafenib); hence, indirect comparisons were possible. Clinical efficacy inputs for the models were derived from the respective, randomized, controlled trials. Three clinicians or oncologists conducted a blinded review of the three aforementioned studies; based on this review, the studies were comparable (Table 1). The specific review content is shown in the appendix Additional file 1: Table S1.

**Table 1:** Comparison of baseline characteristics

	Sorafenib (NCT01761266)		Lenvatinib (NCT01761266)		Atezolizumab- Bevacizumab (NCT03434379)		Sintilimab-Bevacizumab (NCT03794440)	
	N	%	N	%	N	%	N	%
Number of patients	476	100	478	100	336	100	380	100
Age (mean)	64		63		64		53	
Males	401	84	405	85	227	82	334	88
Females	75	16	73	15	109	18	46	12
HBV infection	228	48	251	53	164	49	359	94
HCV infection	126	27	91	19	72	21	6	2
Child–Pugh stage								
A	357	75	368	77	239	72	365	96
B	119	25	110	23	94	28	15	4
BCLC stage								
B	92	19	104	22	52	15	56	15
C	384	81	374	78	276	82	324	85
Presence of macrovascular invasion, extrahepatic metastasis	430	90	441	92	258	77	303	80

*BCLC* barcelona clinic liver cancer, *HBV* hepatitis B virus, *HCV* hepatitis C virus

PFS and OS data for lenvatinib and sorafenib were derived from NCT01761266; PFS and OS data for atezolizumab plus bevacizumab were derived from NCT03434379; and PFS and OS data for sintilimab plus bevacizumab were derived from NCT03794440. Models were extrapolated using the method established by Guyot et al. [13]. The GetData Graph Digitizer, version 2.26,9 software (getdata-graph-digitizer, Inc.) was used to obtain data points from the PFS and OS curves. These data points were subsequently used to fit the following parametric survival functions: Weibull; log-normal; log-logistic; exponential; generalized gamma; and Gompertz. The Bayesian Information Criterion is useful for statistical testing methods [14]. The final survival model selection for each study is shown in Table 2. The validation plots of the subgroups are shown in Additional file 1: Figs. S1–S4.

**Table 2:** Key model inputs

Trial registration identifier	Group	Endpoint	Survival model	Parameter	
				Scale	Shape
NCT01761266	Lenvatinib	PFS	Log-logistic	7.00	1.68
		OS	Log-logistic	13.62	1.77
	Sorafenib	PFS	Log-logistic	4.21	1.56
		OS	Log-logistic	12.52	1.59
NCT03434379	Atezolizumab-Bevacizumab	PFS	Weibull	0.08	1.09
		OS	Log-logistic	21.03	1.32
NCT03794440	Sintilimab-Bevacizumab	PFS	Log-logistic	4.85	1.35
		OS	Log-logistic	15.88	1.71

*OS* overall survival, *PFS* progression-free survival

### Cost and utility inputs

The direct costs that patients need to pay include drugs, testing costs, bed costs, management of adverse reactions, and active treatment after disease progression (Tables 3, 4).The costs are reported in August 2022 US dollars (1 dollar is equal to 6.74 yuan).

**Table 3:** Cost of drug estimates

	Manufacture	Unit cost (US$)	Recommended dosage/frequency	Cost per cycle (21 days) (US$)	Cost per month (30 days) (US$)
Drug therapy costs					
Sorafenib	Bayer	857.14 (60*200 mg)	400 mg twice per day	1200.00	1714.29
Lenvatinib	Eisai	982.56 (30*4 mg)	12 mg per day	2063.37	2947.67
Atezolizumab-Bevacizumab					
Atezolizumab	Roche	4,932.33 (20 ml:1.2 g)	1200 mg per cycle	4932.33	7053.23
Bevacizumab	Roche	225.56 (4 ml:100 mg)	1044 mg per cycle	2481.20	3548.12
Sintilimab-Bevacizumab					
Sintilimab	Xin da	427.52 (10 ml:100 mg)	200 mg per cycle	855.04	1222.70
Bevacizumab	Roche	225.56 (4 ml:100 mg)	1044 mg per cycle	2481.20	3548.12

**Table 4:** Other medical estimates and AE incidences

Other medical costs	Unit cost (US$)	Incidence			
		Sorafenib	Lenvatinib	Atezolizumab-Bevacizumab	Sintilimab-Bevacizumab
General ward	8.4/day	/	/	5 days per cycle	5 days per cycle
Tests ^a^	163.16/set	Once per month	Once per month	Once per month	Once per month
Adverse event	Cost/event				
HFSR	4	12.00%			
Hypertension	38.5		23.00%	15.20%	14%
Subsequent active treatment per patient	1774/month				

*AE* adverse event, *HFSR* hand-foot skin reaction, *MRI* magnetic resonance imagin,*TC* total count

^a^Tests included abdominal ultrasound, MRI, hematological examination, and liver and kidney function. The tests with similar frequency for the two therapies were not included, e.g., TC tests (cost: US$326.31), which were performed once bi-monthly for each therapy

The four treatment regimens in the model followed the dosing intervals and doses provided in their respective labels. In the NCT01761266 trial, the recommended dosage for patients with a body weight < 60 kg and ≥ 60 kg was 8 mg/day and 12 mg/day lenvatinib, respectively; the recommendation for patients who received sorafenib was 400 mg twice daily [7]. It is unfortunately that there is no relevant data to show the average weight of HCC patients in China. After some literature search, they all assumed that the weight of patients with HCC in China is 65 kg [15–17]. According to the data, there are more male than female patients with HCC in China. This model used the average Chinese male weight of 69.6 kg estimated in 2021 to calculate the drug dose [18]. Therefore, in the model, the dosage in the lenvatinib group was 12 mg/day. In the NCT03434379 trial, the recommended dosages were 1,200 mg intravenous atezolizumab and 15 mg/kg bevacizumab once every 3 weeks [8]. In the NCT03794440 trial, patients received 200 mg intravenous sintilimab plus 15 mg/kg bevacizumab, once every 3 weeks [9]. Hence, one treatment cycle required 1,044 mg bevacizumab. The prices of the drugs were obtained from YaoZhi Internet (https://www.yaozh.com/), in which the latest negotiated prices for medicines are occasionally reported.

Except for the drug costs of the four treatment regimens, other costs were calculated based on consultations with physicians from three hospitals; the data provided by the physicians were averaged. Patients on all treatment regimens were followed up once every 2 months. The examinations included abdominal ultrasound, magnetic resonance imaging, blood testing, and liver function testing. Patients receiving intravenous atezolizumab plus bevacizumab or sintilimab plus bevacizumab were required to pay the inpatient bed cost. For all four treatment regimens, costs were calculated for grade 3 adverse reactions with an incidence > 10%, including hand-foot skin reaction and hypertension. Since adverse reactions occurred throughout the course of treatment, the incidence of adverse reactions was allocated evenly to each month.

To ensure model consistency, it was assumed that the four treatment regimens were discontinued at the time of disease progression. Active treatment after progression followed, assuming sequential treatment with regorafenib. For patients experiencing treatment intolerance and progression, the cost of next treatment was US$1,774/month.

The measure utility-specific quality-adjusted life year (QALY) based on the health status was used to determine treatment outcomes. The utility values for PFS, PD, and death were 0.76, 0.68, and 0, respectively (values were derived from Thompson et al.) [19].

### Comparative cost-effectiveness

The cost and utility of the four treatment options were compared using the incremental cost-effectiveness ratio (ICER). According to the recommendations of the World Health Organization and the Guidelines for the Evaluation of Chinese Pharmacoeconomics (2020), this study used three times the per capita GDP of China (US$36,600) reported in 2021 as the willingness-to-pay (WTP) threshold [20–22]. In the comparison with sorafenib, a scheme with an ICER less than WTP is considered to have economic value.

### Perspectives

In China, there is a social insurance and house fund system, which includes medical insurance. Individuals need to pay their contributions on a monthly basis [23]. Through the medical insurance fund, costs related to outpatient visits and hospitalizations can be reimbursed. However, the reimbursement rates differ depending on the diseases and drugs. This complicated process involves medical care diagnosis-related groups/diagnosis-intervention packet payments [24, 25]. In the present study, we analyzed medical care costs from the patient perspective. We considered only the proportion of the patient-copayment for the cost of examination fees, bed fees, adverse reaction processing fees, first-line drug fees, and second-line treatment fees. According to the medical insurance reimbursement policy of Yunnan Province, for ordinary urban residents, the outpatient self-pay rate for sorafenib and lenvatinib was 35% and 40%, respectively. The inpatient self-pay rate for sintilimab plus bevacizumab was approximately 45%. Atezolizumab plus bevacizumab was not covered by the medical insurance reimbursement scheme; hence, the self-pay rate for this regimen was 100% [26].

### Sensitivity analyses

In this study, one-way and probability sensitivity analyses were used to explore the influence of different factors on the results of the model. Upper and lower inputs for one-way sensitivity analyses are shown in Table 5. In one-way sensitivity analyses, the incremental net monetary benefit (INMB) was calculated based on the following formula:

$${\text{INMB}}\left( \lambda \right)\, = \,\left( {\mu _{{{\text{E1}}}} \, - \,\mu _{{{\text{E}}0}} } \right) * \lambda \, - \,\left( {\mu _{{{\text{C1}}}} \, - \,\mu _{{{\text{C}}0}} } \right)\, = \Delta {\text{E}} * \lambda - \,\Delta {\text{C}},$$

where μ_Ei_ and μ_Ci_ are the effectiveness and cost of alternative therapy (i = 1) or sorafenib (i = 0), respectively [27], and λ is three times the GDP per capita of China reported in 2021 (WTP).

**Table 5:** Sensitivity analysis

	One-way sensitivity analysis		PSA	
	Base case value	Range	SD	Distribution
Sorafenib, monthly cost (US$)	1714.29	1371.43–2057.14	342.86	Gamma
Lenvatinib, monthly cost (US$)	2947.67	2358.14–3537.20	589.53	Gamma
Atezolizumab-Bevacizumab, monthly cost (US$)	10,601.35	8481.08–12,721.62	2120.27	Gamma
Sintilimab-Bevacizumab, monthly cost (US$)	4770.82	3816.66–5724.99	954.16	Gamma
Utility PFS	0.76	0.61–0.91	0.15	Normal
Utility PD	0.68	0.54–0.82	0.14	Normal
General ward, cost per cycle (US$)	42.00	33.60–50.40	8.40	Gamma
AE cost	100%	50/200%	75%	Gamma
HCC progression test, cost per test (US$)^a^	163.16	130.53–195.79	32.63	Gamma
Proportion of Atezolizumab-Bevacizumab general ward	100%	50%	N/A	N/A
Proportion of Sintilimab-Bevacizumab general ward	100%	50%	N/A	N/A
Cost of subsequent active treatment per patient (US$)	1774.00	1419.20–2128.80	354.80	Gamma
Discount rate	5%	0–8%	N/A	N/A

*AE* adverse event, *HCC* hepatocellular carcinoma, *N/A* not applicable, *PD* progressive disease, *PFS* progression-free survival, *PSA* probabilistic sensitivity analysis, *SD* standard deviation

^a^Tests included abdominal ultrasound, MRI, hematological examination, and liver and kidney function

The one-way sensitivity analysis was represented using a tornado diagram. The probabilistic sensitivity analysis was performed using Monte Carlo simulation sampling, and the final results were presented using a cost-effectiveness acceptability curve.

## Results

### Cost-effectiveness ranking

**Fig.1:**
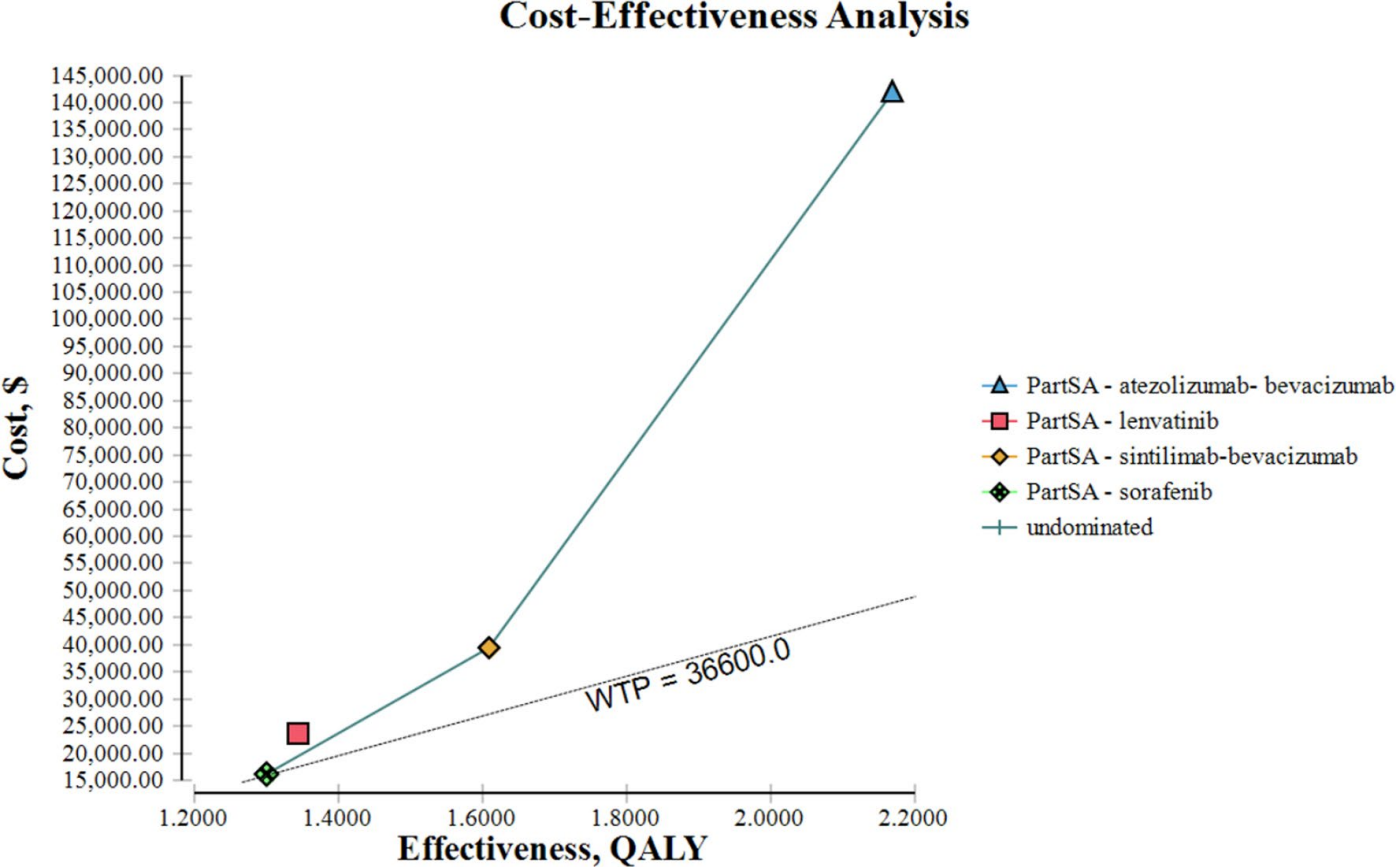
Cost-effectiveness analysis *PartSA* partitioned survival analysis, *QALY* quality adjusted life year, *WTP* willingness-to-pay

From the perspective of the patient, the cost per patient of the sorafenib, lenvatinib, sintilimab plus bevacizumab, and atezolizumab plus bevacizumab treatment regimens after reimbursement by medical insurance was US$16,109.80, US$23,654.81, US$39,406.40, and US$141,836.73, respectively. The QALYs associated with these four therapies was 1.30, 1.34, 1.61, and 2.17, respectively. The cost-effectiveness value of sorafenib was US$12,392.15 per QALY, and the NMB value was US$31,470.20. Compared with lenvatinib, the ICER was US$188,625.25 per QALY, and the NMB value was US$25,389.19. Compared with sorafenib, the ICER of sintilimab plus bevacizumab was US$75,150.32 per QALY, and the NMB value was US$19,519.60. The ICER of atezolizumab plus bevacizumab with sorafenib was US$144,513.71 per QALY, and the NMB value was US$ - 62,414.73. Compared with sorafenib, the ICER of the other three treatment regimens was higher than the WTP threshold (Fig. 1). Table 6 displays the cost-effectiveness ranking of these four therapies.

**Table 6:** Cost-effectiveness ranking per patient (patient perspective)

Strategy	Cost (US$)	Incr cost (US$)	Eff(QALY)	Incr Eff(QALY)	Incr C/E (US$/QALY)	NMB (US$)	INMB (US$)	C/E (US$/QALY)
Sorafenib	16,109.80	0.00	1.30	0.00	0.00	31,470.20	0.00	12,392.15
Lenvatinib	23,654.81	7,545.01	1.34	0.04	188,625.25	25,389.19	− 6,081.01	17,652.84
Sintilimab-Bevacizumab	39,406.40	23,296.60	1.61	0.31	75,150.32	19,519.60	− 11,950.60	24,476.02
Atezolizumab-Bevacizumab	141,836.73	125,726.93	2.17	0.87	144,513.71	− 62,414.73	− 93,884.93	65,362.55

*C/E* cost/effectiveness, *Eff* effectiveness, *Incr* incremental, *INMB* incremental net monetary benefits, *NMB* net monetary benefits

### Sensitivity analysis

**Fig.2:**
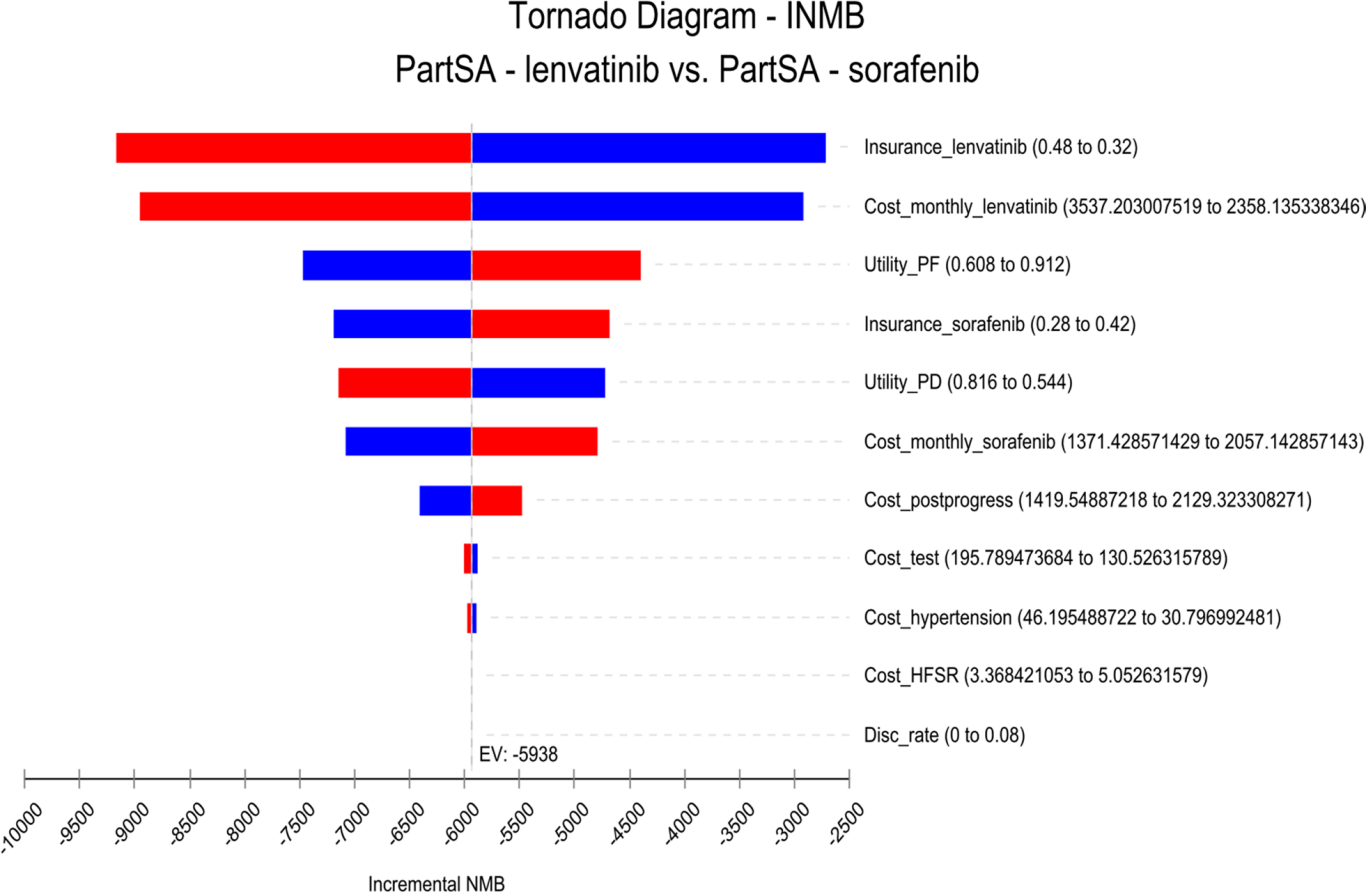
One-way sensitivity analysis of sorafenib versus lenvatinib (INMBs) *disc_rate* discount rate, *HFSR* hand-foot skin reaction, *INMB* incremental net monetary benefits; NMB, net monetary benefits, *PartSA* partitioned survival analysis, *PD* progressive disease, *PF* progression free

**Fig.3:**
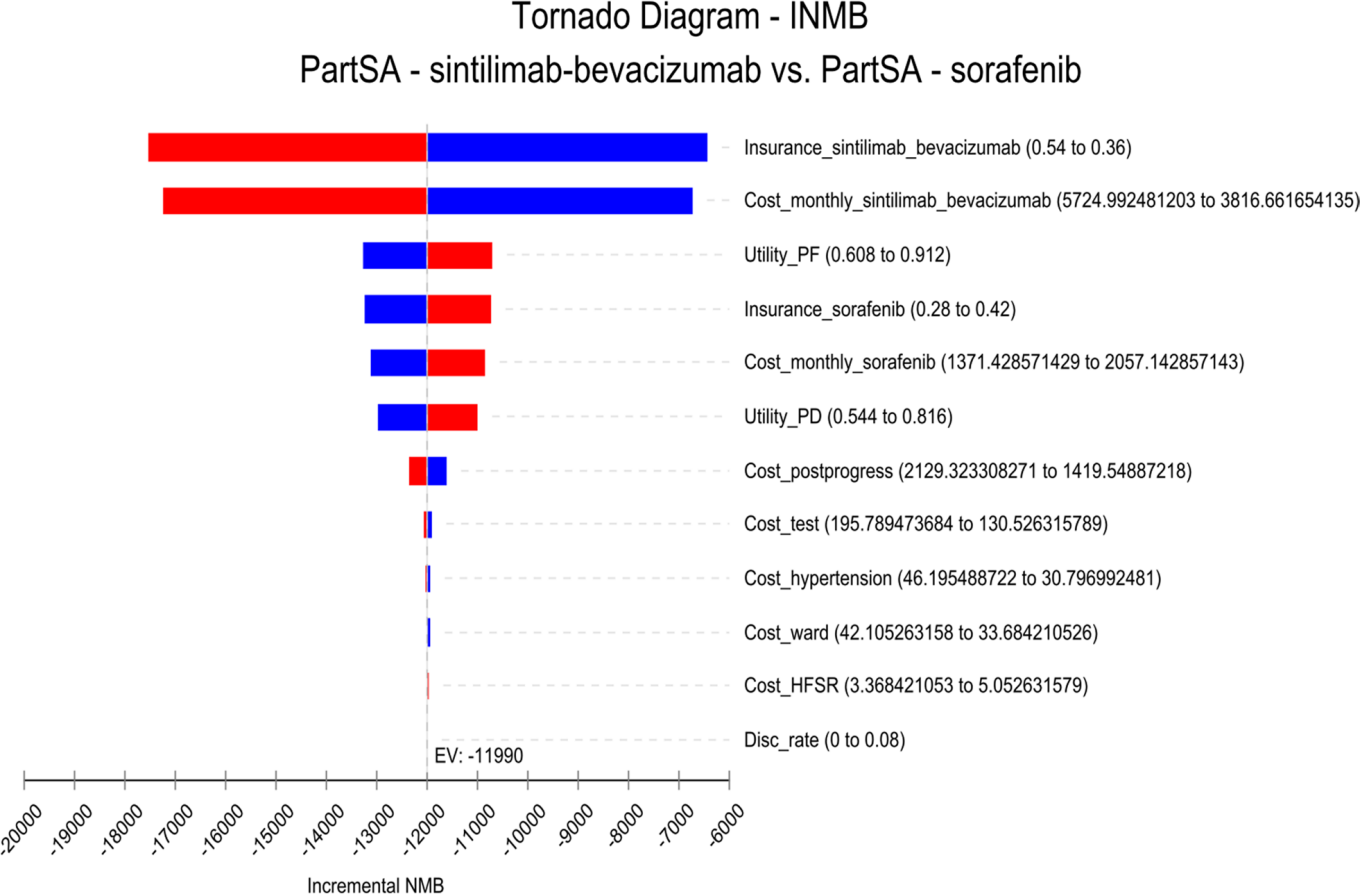
One-way sensitivity analysis of sorafenib versus sintilimab-bevacizumab (INMBs) *disc_rate* discount rate, *HFSR* hand-foot skin reaction, *INMB* incremental net monetary benefits, *NMB* net monetary benefits, *PartSA* partitioned survival analysis, *PD* progressive disease, *PF* progression free

**Fig.4:**
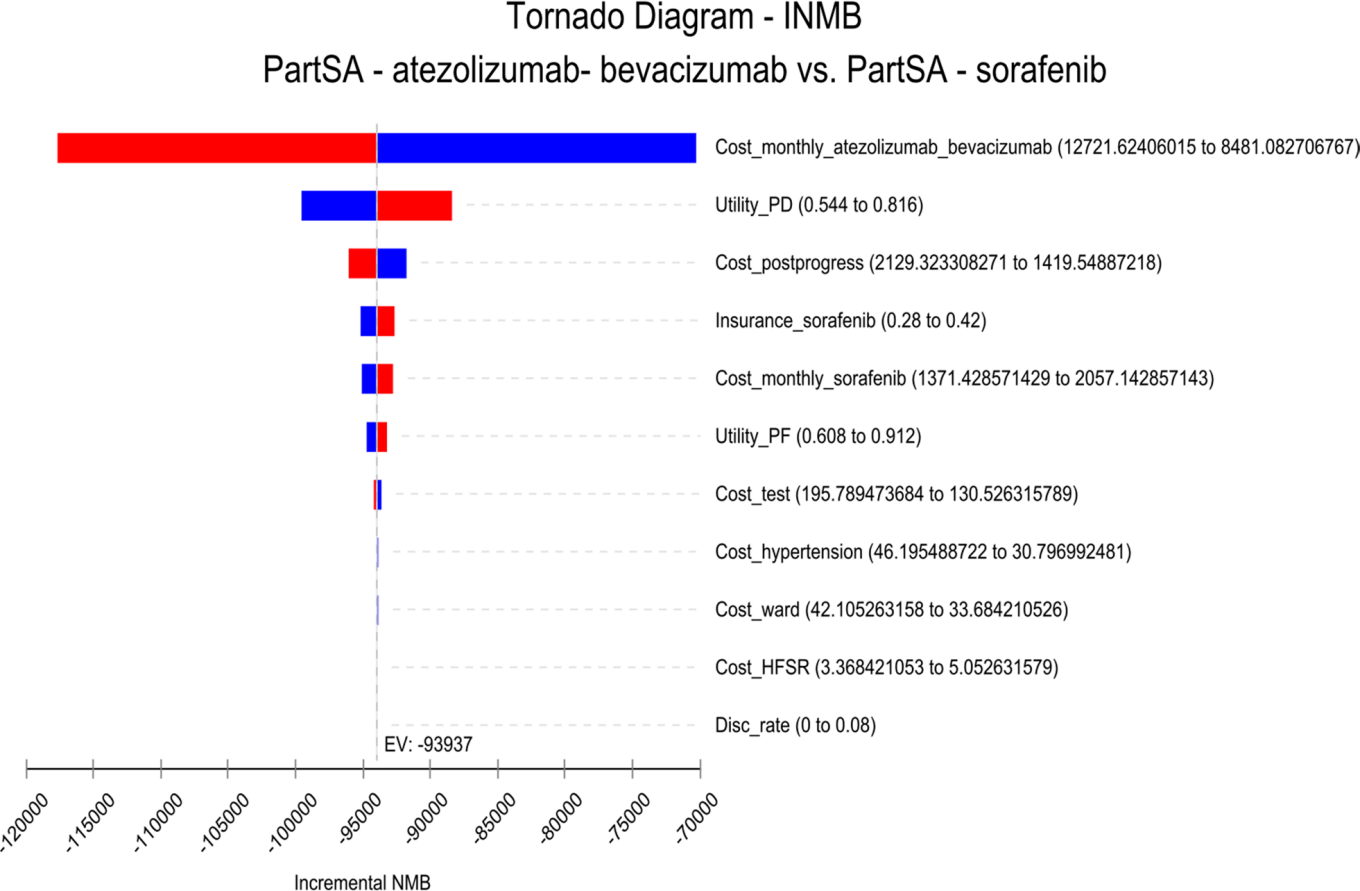
One-way sensitivity analysis of sorafenib versus atezolizumab-bevacizumab (INMBs) disc_rate, discount rate, *HFSR* hand-foot skin reaction, *INMB* incremental net monetary benefits, *NMB* net monetary benefits, *PartSA* partitioned survival analysis, *PD* progressive disease, *PF* progression free

**Fig.5:**
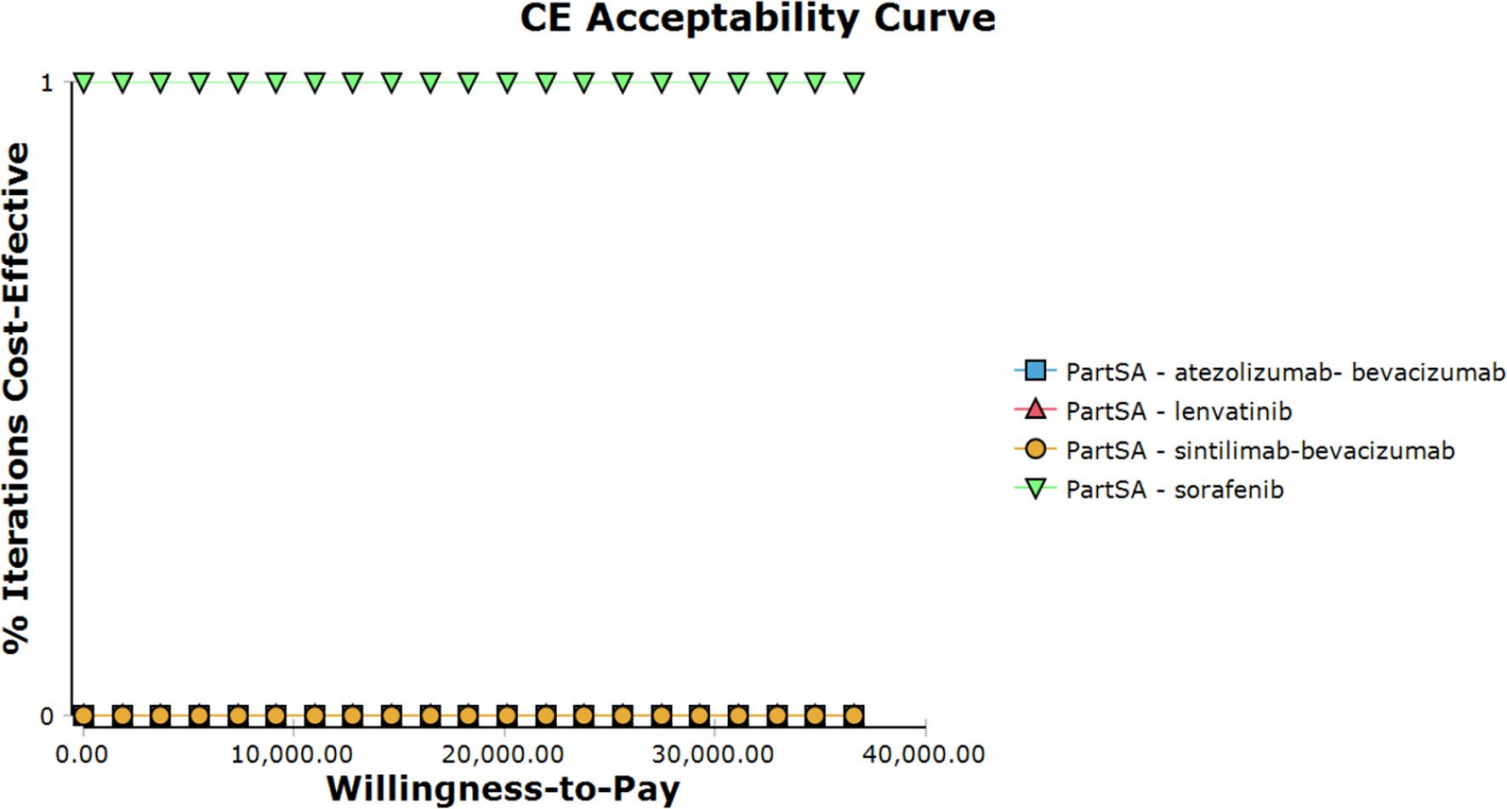
Acceptability curve *CE* cost-effectiveness, *PartSA* partitioned survival analysis

The results of the one-way sensitivity analysis were presented in tornado diagrams (Figs. 2–4). A pairwise comparison of sorafenib and lenvatinib showed that the medical care out-of-pocket ratio and drug price of lenvatinib were the main factors influencing the INMB value. Figure 2 shows that a decrease in the medical care out-of-pocket ratio and drug price of lenvatinib resulted in a gradual increase in the INMB value. A pairwise comparison of sorafenib and sintilimab plus bevacizumab showed that the medical care out-of-pocket ratio and drug price of sintilimab plus bevacizumab were the main factors influencing the INMB value (Fig. 3). The INMB value gradually increased in response to a decrease in the medical care out-of-pocket ratio and drug price of sintilimab plus bevacizumab. Lastly, a pairwise comparison of sorafenib and atezolizumab plus bevacizumab showed that the drug price of atezolizumab plus bevacizumab and the utility of PD were the main factors influencing the INMB value (Fig. 4). As the price of atezolizumab plus bevacizumab decreased, the INMB value gradually increased. However, the INMB value increased simultaneously with the PD value. These data are displayed in the appendix (Additional file 1: Tables S2–S4). The cost-effectiveness acceptability curve was generated to show the probability of cost–utility value. The results showed that only sorafenib is the optimal option when the WTP threshold is US$36,600 (Fig. 5).

## Discussion

Approximately 45% of the global cases of HCC occur in China [1]. HCC can be treated with resective surgery and liver transplantation. However, these options are only available for patients with early-stage HCC and, regrettably, numerous patients are diagnosed with advanced HCC. Prior to 2020, the first-line treatment of advanced HCC was mainly based on two multi-target tyrosine kinase inhibitors, namely sorafenib and lenvatinib. Owing to the recent progress achieved in macromolecular drug research, PD-1 and PD-L1 inhibitors have become the mainstay agents in this setting [28]. It has been shown that targeted monoclonal antibodies effectively prolong the survival of patients with advanced HCC. Currently, there are no studies evaluating the cost-effectiveness of the four aforementioned first-line therapies for advanced HCC in China, an environment with scarce health resources. Therefore, an economic evaluation is warranted to determine the best option for patients by considering both effectiveness and cost.

From the perspective of the patients, the model showed that sorafenib was linked to the smallest value of QALYs per person (i.e., 1.30) among the four treatment regimens. The utility values for lenvatinib, sintilimab plus bevacizumab, and atezolizumab plus bevacizumab were 1.34, 1.61, and 2.17 QALYs, respectively. However, the treatment cost of sorafenib was the lowest among the four regimens (i.e., US$16,109.80 per person). The cost of the other treatment options was US$23,654.81, US$39,406.40, and US$141,836.73, respectively. The ICER for each treatment regimen was obtained through comparison with sorafenib. All ICER values were higher than the WTP threshold, indicating that the three treatment options are not economical. The robustness of the results was confirmed by probabilistic sensitivity analysis. According to the results of the probabilistic sensitivity analysis, 100% of patients selected sorafenib as the treatment regimen, considering a WTP threshold of US$36,600 per QALY.

To our knowledge, this is the first analysis of the latest evidence presented in 2021 by the Chinese Society of Clinical Oncology, through an economic modeling approach to assess treatment cost for advanced HCC. Thus far, few studies investigated the economic outcomes of immune checkpoint inhibitor therapy in advanced HCC [29–31]. In this study, a more systematic and comprehensive economic evaluation of first-line treatment for advanced HCC was conducted.

## Limitations

There were several limitations in the present analysis. Firstly, we calculated the dosages for lenvatinib and bevacizumab using the mean weight of healthy individuals, rather than that of patients with HCC. This is because patients with HCC may have lower weight values, which could affect the estimated dose and cost of lenvatinib and bevacizumab. Notably, this method has been used in another cost-effectiveness model in HCC [29]. Secondly, safety and efficacy data were derived from three independent, randomized, controlled trials. Thus, head-to-head trials of these four first-line regimens in patients with advanced HCC should be conducted to obtain direct evidence on safety and efficacy. Thirdly, this study excluded the treatment costs for grades 1–2 adverse reactions, which may have led to bias. This limitation may not be a major factor, as the results of the one-way sensitivity analysis suggested that costs associated with adverse events are minimal. Finally, the proportion of out-of-pocket payments from the perspective of patients was calculated in accordance with the relevant policies of medical insurance in Yunnan Province. However, the reimbursement policies of medical insurance differ between provinces in China. Thus, the conclusions drawn in this study may not be applicable to other regions. Nonetheless, the results of this assessment reflect general clinical practice for the management of advanced HCC. Hence, the present evidence may be of value to physicians and policymakers.

## Conclusion

The present study showed that lenvatinib, sintilimab plus bevacizumab, and atezolizumab plus bevacizumab are not cost-effective first-line options for the treatment of unresectable HCC from the perspective of the patient. Nevertheless, the cost associated with these three treatment regimens may be reduced through a reduction in drug costs and adjustment of medical care reimbursement policies. The findings of this study may facilitate clinical treatment planning for patients with advanced HCC.

## Supplementary Information

**Additional file 1: Table S1**. Inclusion and exclusion criteria of the NCT01761266, NCT03434379 and NCT03794440 studies. **Table S2.** One-way sensitivity analysis of sorafenib and lenvatinib, discounted (per patient). **Table S3.** One-way sensitivity analysis of sorafenib and sintilimab–bevacizumab, discounted (per patient). **Table S4.** One-way sensitivity analysis of sorafenib and atezolizumab–bevacizumab, discounted (per patient). **Figure S1.** The Replicated Kaplan-Meier survival Curves of sorafenib in NCT01761266 Trial. **Figure S2.** The Replicated Kaplan-Meier survival Curves of lenvatinib in NCT01761266 Trial. **Figure S3.** The Replicated Kaplan-Meier survival Curves of S+B in NCT03794440 Trial. **Figure S4.** The Replicated Kaplan-Meier survival Curves of A+B in NCT03434379 Trial. **Figure S5.** One-way sensitivity analysis of ICER of sorafenib and lenvatinib, discounted (per patient), payer system perspective. **Figure S6.** One-way sensitivity analysis of ICER of sorafenib and sintilimab–bevacizumab, discounted (per patient), payer system perspective.

## Data Availability

The datasets generated during and/or analysed during the current study are available from the first author on reasonable request.

## Code availability

The code generated during and/or analysed during the current study are available from the first author on reasonable request.
