# Supplementary material for: Cost-effectiveness analysis of sorafenib, lenvatinib, atezolizumab plus bevacizumab and sintilimab plus bevacizumab for the treatment of advanced hepatocellular carcinoma in China
Source: Cost Eff Resour Alloc. 2023 Mar 31;21:20. doi: 10.1186/s12962-023-00435-x (PMC10064722; doi:10.1186/s12962-023-00435-x)
Supplement: Supplementary file 1 — Additional file 1: Table S1. Inclusion and exclusion criteria of the NCT01761266, NCT03434379 and NCT03794440 studies. Table S2. One-way sensitivity analysis of sorafenib and lenvatinib, discounted (per patient). Table S3. One-way sensitivity analysis of sorafenib and sintilimab–bevacizumab, discounted (per patient). Table S4. One-way sensitivity analysis of sorafenib and atezolizumab–bevacizumab, discounted (per patient). Figure S1. The Replicated Kaplan-Meier survival Curves of sorafenib in NCT01761266 Trial. Figure S2. The Replicated Kaplan-Meier survival Curves of lenvatinib in NCT01761266 Trial. Figure S3. The Replicated Kaplan-Meier survival Curves of S+B in NCT03794440 Trial. Figure S4. The Replicated Kaplan-Meier survival Curves of A+B in NCT03434379 Trial. Figure S5. One-way sensitivity analysis of ICER of sorafenib and lenvatinib, discounted (per patient), payer system perspective. Figure S6. One-way sensitivity analysis of ICER of sorafenib and sintilimab–bevacizumab, discounted (per patient), payer system perspective. [file 12962_2023_435_MOESM1_ESM.docx]

Supplementary Material

Cost-effectiveness analysis of sorafenib, lenvatinib, atezolizumab plus bevacizumab and sintilimab plus bevacizumab for the treatment of advanced hepatocellular carcinoma in China

Gong Hongyu1,2, Ph.D. candidate, Teaching assistant

ONG Siew Chin1*, MD, Professor

Li Fan3, MD, Professor

Zhao Keying3, MD, Associate professor

Weng Zhiying3, MD, Professor

Jiang Zhengyou4, Ph.D. candidate

^1^School of Pharmaceutical Sciences, Universiti Sains Malaysia, 11800 USM Penang, Penang City, Malaysia

^2^Incubation Center for Scientific and Technological Achievements, Kunming Medical University, Chunrong west road 1168, Kunming City, China

^3^School of Pharmaceutical Science &Yunnan Key Laboratory of Pharmacology for

Natural Products, Kunming Medical University, Chunrong west road 1168, Kunming City, China

^4^School of Public Health, Kunming Medical University, Chunrong west road 1168, Kunming City, China

^5^School of management, Universiti Sains Malaysia, 11800 USM Penang, Penang City, Malaysia

*** Correspondence author:**

ONG Siew Chin

siewchinong@usm.my

Universiti Sains Malaysia,

Phone: 604-6533888 Ext: 4725 (office)

**Table S1 Inclusion and exclusion criteria of the NCT01761266, NCT03434379 and NCT03794440 studies**

| Criteria | NCT01761266 | NCT03434379 | NCT03794440 |
| --- | --- | --- | --- |
| Age requirements | ≥18 | ≥18 | ≥18 |
| Previous treatment | No received previous systemic therapy | No previously received systemic therapy | No previous systemic therapy |
| How HCC diagnosed | Histologically or cytologically, or confirmed clinically in accordance with American Association for the Study of Liver Diseases criteria | Histologic or cytologic analysis or clinical features according to the American Association for the Study of Liver Diseases criteria for patients with cirrhosis | Histologically, cytologically, or clinically confirmed per the American Association for the Study of Liver Disease criteria |
| Lesions | One or more measurable [target lesions](https://www.sciencedirect.com/topics/medicine-and-dentistry/target-lesion) | N/A | One or more measurable target lesions |
| Life expectancy | 12 weeks+ | N/A | 12 weeks+ |
| BCLC stage | B or C | N/A | B or C |
| ECOG | 0 or 1 | 0 or 1 | 0 or 1 |
| Child-Pugh liver function | A | A | ≤7 |
| Exclusion criteria | With 50% or higher liver occupation, obvious invasion of the bile duct, or invasion at the main portal vein | A history of autoimmune disease；high risk of bleeding | A history of autoimmune  disease,liver transplantation, acute or chronic active HBV  or HCV infection |

Abbreviations: BCLC stage, barcelona clinic liver cancer stage; ECOG, Eastern Cooperative Oncology Group; HCC, Hepatocellular carcinoma; HBV, hepatitis B virus; HCV, hepatitis C virus；

**Figure S1:The Replicated Kaplan-Meier survival Curves of sorafenib in NCT01761266 Trial**


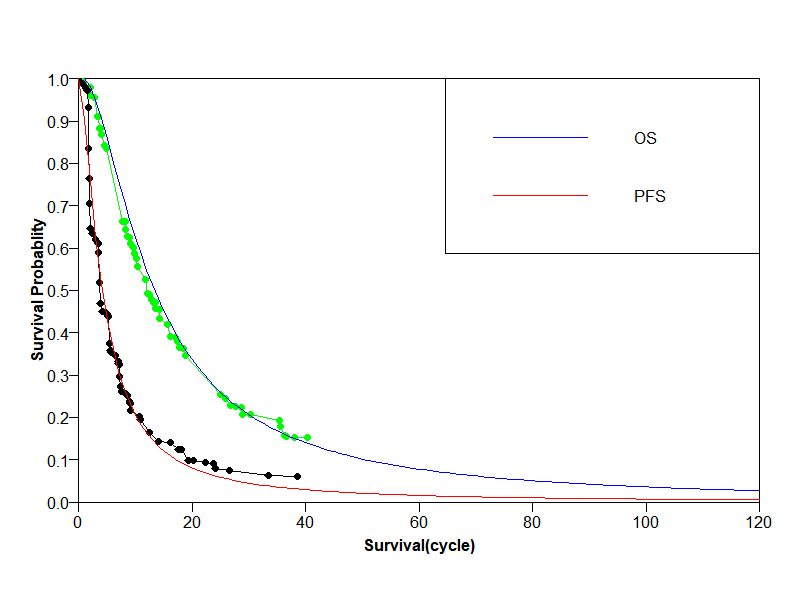


Abbreviations: PFS, progression-free survival; OS, overall survival

**Figure S2:The Replicated Kaplan-Meier survival Curves of lenvatinib in NCT01761266 Trial**


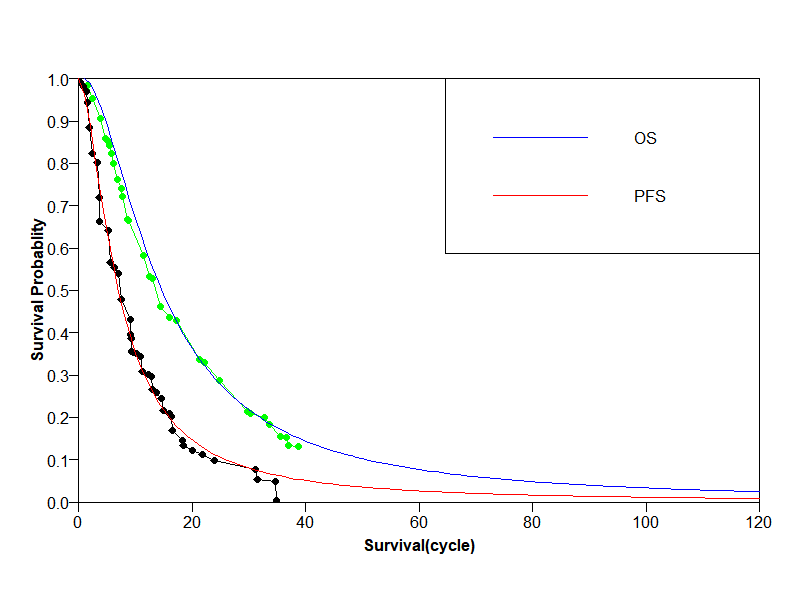


Abbreviations: PFS, progression-free survival; OS, overall survival

**Figure S3:The Replicated Kaplan-Meier survival Curves of S+B in NCT03794440 Trial**


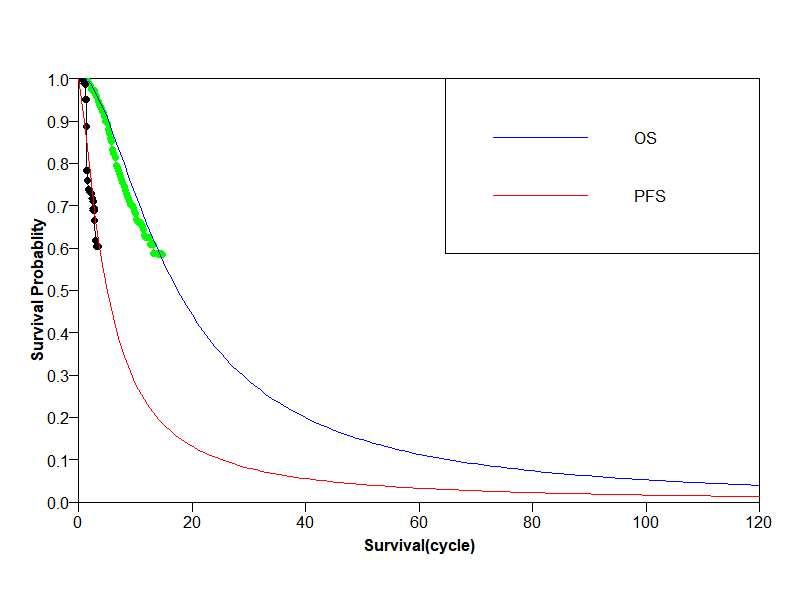


Abbreviations: PFS, progression-free survival; OS, overall survival

**Figure S4:The Replicated Kaplan-Meier survival Curves of A+B in NCT03434379 Trial**


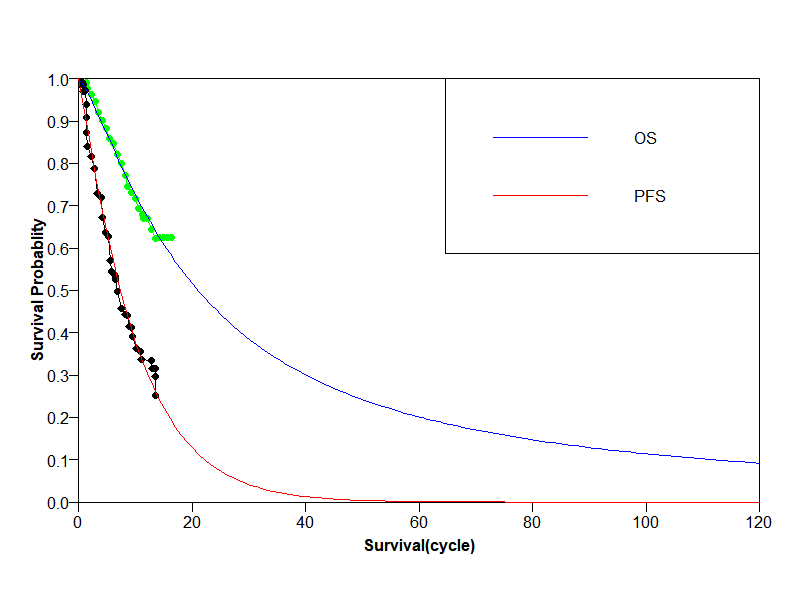


Abbreviations: PFS, progression-free survival; OS, overall survival；

**Table S2：One-way sensitivity analysis of sorafenib and lenvatinib, discounted (per patient)**

| **Variable Name** | **Variable Low** | **Variable Base** | **Variable High** | **Impact** | **Low-INMB** | **High-INMB** |
| --- | --- | --- | --- | --- | --- | --- |
| insurance_lenvatinib | 0.32 | 0.40 | 0.48 | Decrease | -9164.48 | -2710.55 |
| cost_monthly_lenvatinib | 2358.14 | 2947.67 | 3537.20 | Decrease | -8957.86 | -2917.17 |
| util_PF | 0.61 | 0.76 | 0.91 | Increase | -7470.95 | -4404.08 |
| insurance_sorafenib | 0.28 | 0.35 | 0.42 | Increase | -7188.88 | -4686.15 |
| util_PD | 0.54 | 0.68 | 0.82 | Decrease | -7149.45 | -4725.58 |
| cost_monthly_sorafenib | 1371.43 | 1714.29 | 2057.14 | Increase | -7077.58 | -4797.45 |
| cPostProgress | 1419.55 | 1774.44 | 2129.32 | Increase | -6404.11 | -5470.92 |
| cost_test | 130.53 | 163.16 | 195.79 | Decrease | -5996.19 | -5878.84 |
| cost_hypertension | 30.80 | 38.50 | 46.20 | Decrease | -5976.96 | -5898.07 |
| cost_HFSR | 3.37 | 4.21 | 5.05 | Increase | -5940.32 | -5934.72 |
| disc_rate | 0.00 | 0.05 | 0.08 | Increase | -5937.52 | -5937.52 |

Abbreviations: PFS, progression-free survival; PD, progressed disease; HFSR, hand-foot skin reaction; disc_rate, discount rate;

**Table S3：One-way sensitivity analysis of sorafenib and sintilimab–bevacizumab, discounted (per patient)**

| **Variable Name** | **Variable Low** | **Variable Base** | **Variable High** | **Impact** | **Low-INMB** | **High-INMB** |
| --- | --- | --- | --- | --- | --- | --- |
| insurance_sintilimab_  bevacizumab | 0.36 | 0.45 | 0.54 | Decrease | -17522.29 | -6457.72 |
| cChemo_monthly_sintilimab_bevacizumab | 3816.66 | 4770.83 | 5724.99 | Decrease | -17253.37 | -6726.64 |
| util_PFS | 0.61 | 0.76 | 0.91 | Increase | -13268.47 | -10711.54 |
| insurance_sorafenib | 0.28 | 0.35 | 0.42 | Increase | -13241.37 | -10738.64 |
| cost_monthly_sorafenib | 1371.43 | 1714.29 | 2057.14 | Increase | -13130.07 | -10849.94 |
| util_PD | 0.54 | 0.68 | 0.82 | Increase | -12972.86 | -11007.15 |
| cost_PostProgress | 1419.55 | 1774.44 | 2129.32 | Decrease | -12368.41 | -11611.60 |
| cost_test | 130.53 | 163.16 | 195.79 | Decrease | -12061.50 | -11918.51 |
| cost_hypertension | 30.80 | 38.50 | 46.20 | Decrease | -12032.47 | -11947.53 |
| cost_HFSR | 3.37 | 4.21 | 5.05 | Increase | -11992.80 | -11987.20 |
| disc_rate | 0.00 | 0.05 | 0.08 | Increase | -11990.00 | -11990.00 |

Abbreviations: PFS, progression-free survival; PD, progressed disease; HFSR, hand-foot skin reaction; disc_rate, discount rate;

**Table S4：One-way sensitivity analysis of sorafenib and atezolizumab–bevacizumab, discounted (per patient)**

| **Variable Name** | **Variable Low** | **Variable Base** | **Variable High** | **Impact** | **Low-INMB** | **High-INMB** |
| --- | --- | --- | --- | --- | --- | --- |
| cost_monthly_atezolizumab_bevacizumab | 8481.08 | 10601.35 | 12721.62 | Decrease | -117640.11 | -70233.05 |
| utility_PD | 0.54 | 0.68 | 0.82 | Increase | -99516.28 | -88356.88 |
| cost_PostProgress | 1419.55 | 1774.44 | 2129.32 | Decrease | -96084.78 | -91788.38 |
| insurance_sorafenib | 0.28 | 0.35 | 0.42 | Increase | -95187.95 | -92685.21 |
| cost_monthly_sorafenib | 1371.43 | 1714.29 | 2057.14 | Increase | -95076.64 | -92796.52 |
| utility_PFS | 0.61 | 0.76 | 0.91 | Increase | -94714.95 | -93158.21 |
| cost_test | 130.53 | 163.16 | 195.79 | Decrease | -94192.88 | -93680.28 |
| cost_hypertension | 30.80 | 38.50 | 46.20 | Decrease | -94022.66 | -93850.51 |
| cost_HFSR | 3.37 | 4.21 | 5.05 | Increase | -93939.38 | -93933.78 |
| disc_rate | 0.00 | 0.05 | 0.08 | Increase | -93936.58 | -93936.58 |

Abbreviations: PFS, progression-free survival; PD, progressed disease; HFSR, hand-foot skin reaction; disc_rate, discount rate;

**Figure S5：One-way sensitivity analysis of ICER of sorafenib and lenvatinib, discounted (per patient), payer system perspective**


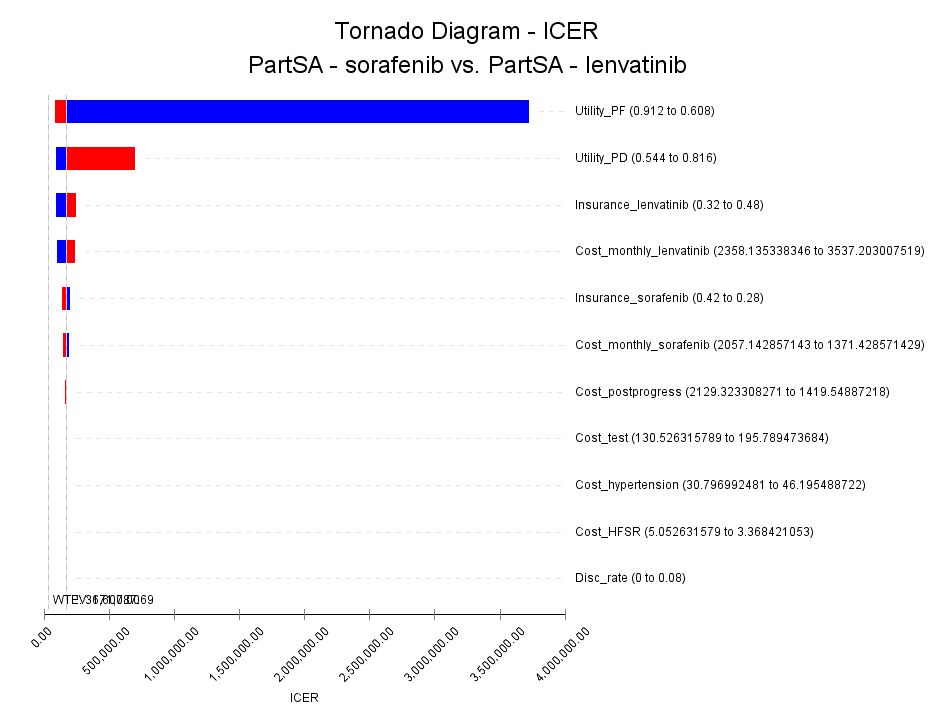


Abbreviations: PartSA, partitioned survival analysis; ICER, incremental cost-effectiveness ration; PF, progression free; PD, progressed-disease; HFSR, hand-foot skin reaction; disc_rate, discount rate;

**Figure S6：One-way sensitivity analysis of ICER of sorafenib and sintilimab–bevacizumab, discounted (per patient), payer system perspective**


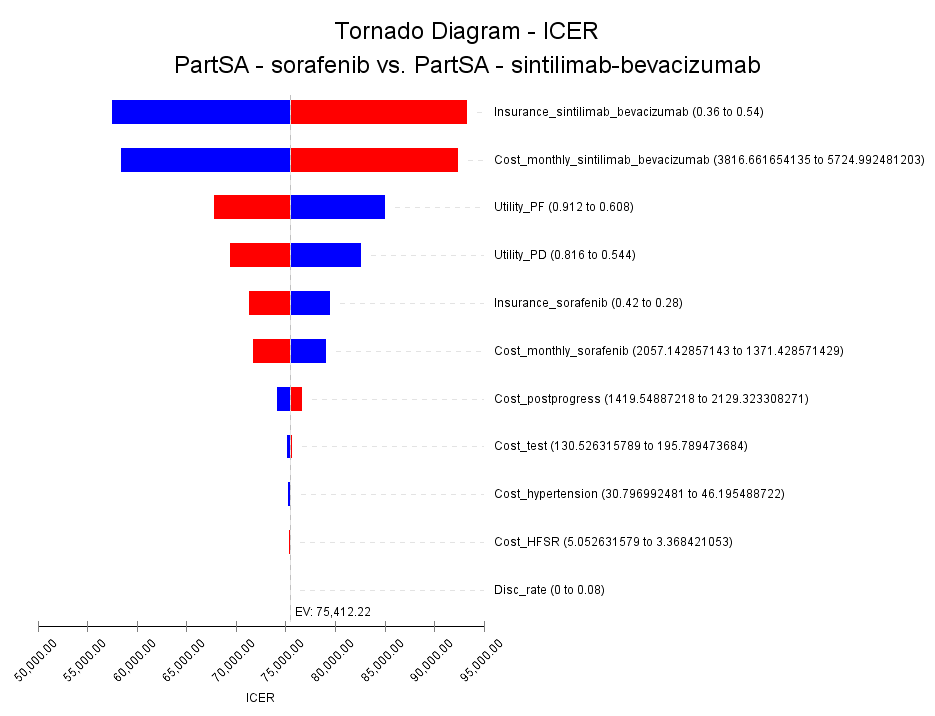


Abbreviations: PartSA, partitioned survival analysis; ICER, incremental cost-effectiveness ration; PF, progression free; PD, progressed-disease; HFSR, hand-foot skin reaction; disc_rate, discount rate;

**Figure S7：One-way sensitivity analysis of ICER of sorafenib and atezolizumab–bevacizumab, discounted (per patient), payer system perspective**


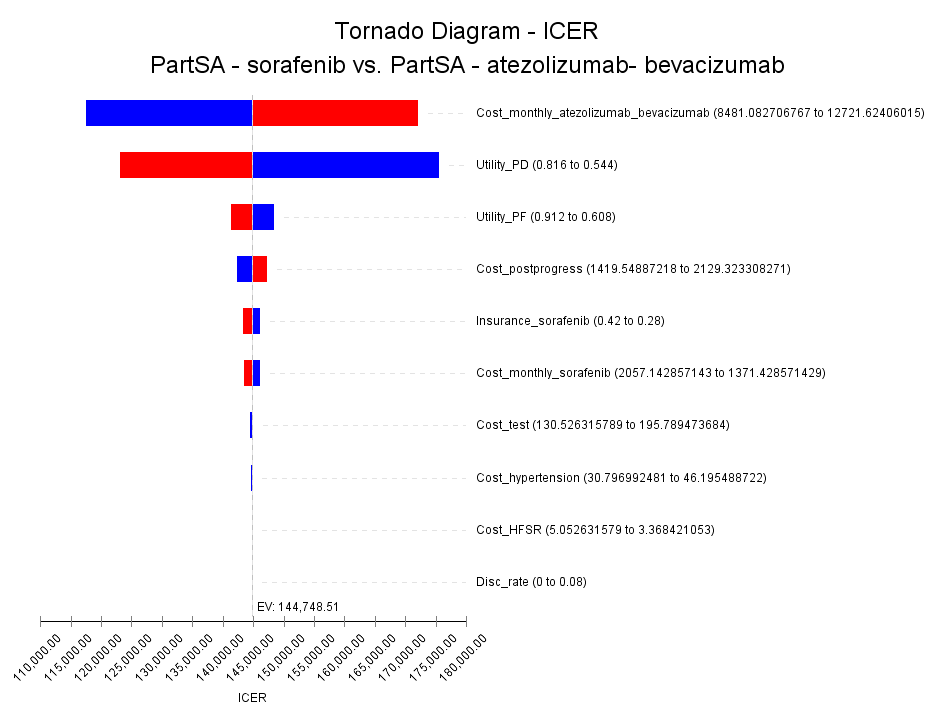


Abbreviations: PartSA, partitioned survival analysis; ICER, incremental cost-effectiveness ration; PF, progression free; PD, progressed-disease; HFSR, hand-foot skin reaction; disc_rate, discount rate;
